# Prolonged warm ischemia time leads to severe renal dysfunction of donation-after-cardiac death kidney grafts

**DOI:** 10.1038/s41598-021-97078-w

**Published:** 2021-09-09

**Authors:** Peter Urbanellis, Laura Mazilescu, Dagmar Kollmann, Ivan Linares-Cervantes, J. Moritz Kaths, Sujani Ganesh, Fabiola Oquendo, Manraj Sharma, Toru Goto, Yuki Noguchi, Rohan John, Ana Konvalinka, Istvan Mucsi, Anand Ghanekar, Darius Bagli, Lisa A. Robinson, Markus Selzner

**Affiliations:** 1 Soham and Shaila Ajmera Family Transplant Centre, University of Toronto General Surgery and Multi-Organ Transplant Program, Toronto General Hospital, University Health Network, 585 University Avenue, 11 PMB-178, Toronto, ON M5G 2N2 Canada; 2 Canadian Donation and Transplantation Research Program, Edmonton, AB Canada; 3 Institute of Medical Science, University of Toronto, Toronto, ON Canada; 4 General, Visceral and Transplantation Surgery, University Hospital Essen, Essen, Germany; 5 Department of General Surgery, Medical University of Vienna, Vienna, Austria; 6 Laboratory Medicine and Pathobiology, Toronto General Hospital, University of Toronto, Toronto, ON Canada; 7 Department of Medicine, Division of Nephrology, University Health Network, Toronto, ON Canada; 8 Departments of Surgery (Urology) and Physiology, The Hospital for Sick Children, University of Toronto, Toronto, ON Canada; 9 Program in Developmental and Stem Cell Biology, The Hospital For Sick Children Research Institute, Toronto, ON Canada; 10 Division of Nephrology, The Hospital for Sick Children, 555 University Avenue, Toronto, ON M5G 1X8 Canada; 11 Program in Cell Biology, The Hospital for Sick Children Research Institute, Toronto, ON Canada

**Keywords:** Acute inflammation, Translational research, Kidney diseases

## Abstract

Kidney transplantation with grafts procured after donation-after-cardiac death (DCD) has led to an increase in incidence of delayed graft function (DGF). It is thought that the warm ischemic (WI) insult encountered during DCD procurement is the cause of this finding, although few studies have been designed to definitely demonstrate this causation in a transplantation setting. Here, we use a large animal renal transplantation model to study the effects of prolonged WI during procurement on post-transplantation renal function. Kidneys from 30 kg-Yorkshire pigs were procured following increasing WI times of 0 min (Heart-Beating Donor), 30 min, 60 min, 90 min, and 120 min (n = 3–6 per group) to mimic DCD. Following 8 h of static cold storage and autotransplantation, animals were followed for 7-days. Significant renal dysfunction (SRD), resembling clinical DGF, was defined as the development of oliguria < 500 mL in 24 h from POD3-4 along with POD4 serum potassium > 6.0 mmol/L. Increasing WI times resulted in incremental elevation of post-operative serum creatinine that peaked later. DCD120min grafts had the highest and latest elevation of serum creatinine compared to all groups (POD5: 19.0 ± 1.1 mg/dL, *p* < 0.05). All surviving animals in this group had POD4 24 h urine output < 500 cc (mean 235 ± 172 mL) and elevated serum potassium (7.2 ± 1.1 mmol/L). Only animals in the DCD120min group fulfilled our criteria of SRD (*p* = 0.003), and their renal function improved by POD7 with 24 h urine output > 500 mL and POD7 serum potassium < 6.0 mmol/L distinguishing this state from primary non-function. In a transplantation survival model, this work demonstrates that prolonging WI time similar to that which occurs in DCD conditions contributes to the development of SRD that resembles clinical DGF.

## Introduction

The success of kidney transplantation for end-stage kidney disease (ESKD) is limited by the profound shortage of available donor kidneys, with approximately 6% of all transplant candidates dying on the waitlist worldwide^1^. This has led to an increase in the use of donation-after-cardiac death (DCD) grafts^2^.

DCD grafts are procured following the withdrawal of mechanical supports (WMS) in the presence of brainstem reflexes. Following WMS, the donor enters the agonal phase until death is determined. The length of the agonal phase is highly variable and often characterized by relative hypotension and/or hypoxemia that can lead to poor perfusion/oxygenation of the donor kidney^3^. To limit transplantation of irreversibly injured grafts, transplant programs require donors to be pronounced dead within a set timeframe, typically within 120 min from WMS. Additionally, procurement does not occur for 2-5 min after the determination of death to ensure that autoresuscitation will not occur^3^. The possible warm ischemic injury encountered following the time from WMS results in concerns unique to DCD grafts.

Delayed graft function (DGF) is more common in DCD vs donation after neurological death kidney transplantation and it occurs in approximately 50% of DCD grafts^4^. No standardized definition of DGF exists, however the requirement for dialysis within the first week following transplantation is most commonly accepted. Alternative definitions include less than 25% decrease in recipient serum creatinine (SCr) 24 h after kidney transplantation or a failure of a 10% decrease in SCr for 3 consecutive days within the first week after transplantation^4^. DGF grafts function after a recovery period, which is in contrast to primary non-function (PNF) with a permanent need for dialysis.

DGF has been correlated with many adverse effects. Immediately post-transplantation, it results in decreased patient quality-of-life, additional evaluation with invasive biopsies, the need for dialysis, prolonged hospital stays, and associated increase in health care costs^5,6^. Large registry studies have also associated the presence of DGF with an increase in episodes of acute rejection and a decrease in long-term graft survival and function^6–8^.

Many graft and recipient factors have been identified as potential contributors to the development of DGF in DCD kidneys. This includes donor age, body-mass index, history of hypertension, and last SCr prior to procurement^7^. Human Leukocyte Antigen mismatches and recipient age, sex, and diagnosis of diabetes have also been identified as important factors. Prolonged ischemic time in DCD conditions have been correlated with DGF, however mechanistic studies demonstrating this causation utilizing renal DCD transplantation survival models are lacking^9^.

This study addresses this gap in knowledge by utilizing a large animal renal DCD autotransplantation model and prolonging WI time prior to kidney procurement. Here, we also demonstrate that extending WI time leads to the development of significant renal dysfunction (SRD) that resembles the clinical development of DGF. A reproducible and clinically relevant animal model of SRD is important to allow for the study of pathophysiological mechanisms that contribute to DGF as well as to study therapeutic strategies to decrease its occurrence.

## Materials and methods

### Animals

Yorkshire pigs at 30 kg were purchased and delivered from Caughell farms (Fingal, Ontario). The husbandry and experimental protocols were approved by our Toronto General Hospital institutional research ethics board. The care of these animals followed recommendations from the *Principles of Laboratory Animal Care* by the National Society for Medical Research and the *Guide for the Care of Laboratory Animals* by the National Institutes of Health. This study was carried out in compliance with the ARRIVE guidelines (https://arriveguidelines.org). While maintained in species-adapted housing, water and food were provided ad libitum*.*

### Experimental design

**Figure 1:**
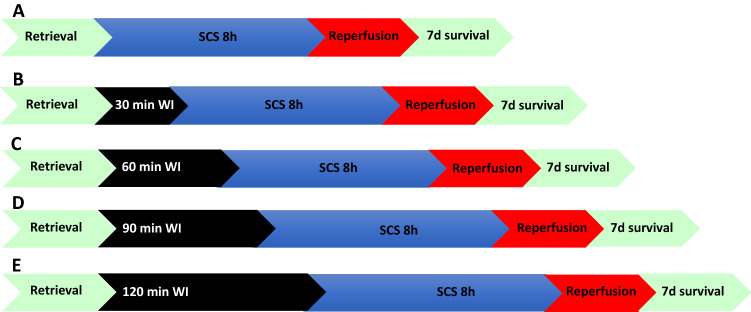
Experimental groups and design. Prior to procurement, animals were subjected to (**A**) 0 min (heart beating donors, n = 3), (**B**) 30 min (n = 5), (**C**) 60 min (n = 3), (**D**) 90 min (n = 3), or (**E**) 120 min (n = 6) of warm ischemia to mimic donation-after cardiac death. Animals were observed for 7 days after transplantation. *SCS* Static Cold Storage; *WI* Warm ischemia.

DCD conditions were mimicked in the porcine setting by clamping renal vessels prior to procurement. Warm ischemia (WI) times were progressively increased to create the following groups: HBD (heart-beating donor) with 0 min WI (n = 3)^10^, DCD30min with 30 min WI (n = 5)^11^, DCD60min with 60 min WI (n = 3), DCD90min with 90 min WI (n = 3), DCD120min with 120 min WI (n = 6). All groups were then stored in static cold storage (SCS) for 8 h (Fig. 1).

### Kidney retrieval: the DCD procurement model

Our laboratory previously described our porcine kidney retrieval strategy^12^. Animals are first provided anesthesia through intramuscular injections of midazolam (0.3 mg/kg; Pharmaceutical Partners of Canada Inc, Richmond Hill, Canada), ketamine (20 mg/kg; Bimeda-MTC Animal Health Inc., Cambridge, Canada), and atropine (0.04 mg/kg; Rafter 8 Products, Calgary, Canada). Following intubation with a 6.5Fr endotracheal tube (Covidien, Brampton, Canada), isoflurane (1.5%; Pharmaceutical Partners of Canada Inc; Richmond Hill, Canada) is provided for maintenance anesthesia.

Sterile technique was maintained throughout the procedure. While supine, a midline incision was made with the right kidney exposed and dissected. In appropriate groups, heparin (2,000 international units; Sandoz Canada Inc., Toronto, Canada) was administered prior to clamping of renal vessels. Following right kidney resection, the renal artery was cannulated, and the kidney was immediately flushed with 300 mL of histidine-tryptophan-ketoglutarate (HTK) solution (Metapharm Inc., Brantford, Canada) at 4 °C. Kidneys were then placed for 8 h in static cold storage (SCS) conditions consisting of HTK solution in an organ bag stored on ice. The abdomen was closed, and animals recovered from anesthesia during this storage time.

### Kidney transplantation

Kidney transplantation was performed as previously described by our group by multiple primary surgeons^12^. In short, animals were re-anesthetized following kidney storage with a 5 mL bolus of propofol delivered intravenously (IV) (PharmaScience Inc., Montreal, Canada) followed by continuous IV propofol administration at 150 mg/h. After re-intubation, 1.5% isofluorane was provided. The midline laparotomy incision was re-opened following cleansing and draping of the site. The left kidney was dissected and discarded. The stored kidney was flushed with 300 mL of HTK prior to transplantation with the following anastomoses: renal vein end-to-side to vena cava, renal artery end-to-side to aorta, and donor ureter side-to-side to recipient ureter.

Postoperative care, drug, and antibiotic administration along with blood collection were performed as previously described^12^. Animals were followed for 7 days following transplantation.

### Whole blood, serum, and urine analysis

Venous blood gas analysis (RAPIDPoint 500 Systems; Siemens AG, Berlin, Germany) was performed immediately following central line placement and daily following transplantation. Using venous blood samples, post-operative measurements of SCr and electrolytes were performed daily (Piccolo Xpress, Union City, Canada).

On POD3-4 and on POD6-7, animals were placed in a custom-designed cage where urine funnels through a filter and is collected for 24 h for calculation of creatinine clearance (CrCl).

### Histology

POD7 renal biopsies were collected by wedge resection with the animal under anesthesia. Tissue was placed in 10% neutral buffered formalin and transferred to 70% ethanol after 48 h. After paraffin-embedding, sectioning, and staining, 3 µm periodic acid-Schiff (PAS)-stained sections were used to score tubular injury and interstitial inflammation on a scale of 0-to-3, respectively, by a single pathologist blinded to the experimental group^13,14^. Tubular injury, including brush border loss, tubular dilation, epithelial vacuolation, thinning and sloughing, and luminal debris were scored in 10 high-power fields and averaged to assess overall tubular injury. Interstitial inflammation was scored in 10 low-power fields and averaged^13,14^.

### Definition of significant renal dysfunction (SRD)

DGF is most commonly defined as the need for dialysis within the first week after kidney transplantation^4^. Dialysis is not feasible in pigs. Our a priori definition of SRD resembling DGF in the porcine setting is the indication for dialysis within the first post-operative week. Specifically, we defined SRD as a serum potassium above 6.0 mmol/L on the 4th post-operative day together with a urine output (UO) < 500 mL in the accompanying 24 h period. Day 4 was chosen as this was the earliest timepoint we performed 24 h urine collection with pigs in the custom-designed cage. Ethical and logistical considerations precluded continuous urine collection. Oliguria is not defined in pigs. Normal UO of 12-19 kg of Danish Landrace pigs at 9-13 weeks of age in a 24 h period has been reported at 2845 ± 900mL^15,16^. Although our pigs are larger (approximately 30 kg), we choose 500 mL as low UO as this represents a convenient threshold that is more than 2.5 standard deviations from this mean.

### Statistical analysis

Significance was defined as *p* < 0.05. A log-rank test was used for calculation of differences in mortality. ANOVA analysis with post-hoc Tukey’s honestly significant difference test was used to identify significance in normally distributed continuous parameters between multiple groups. Binomial categorical data were assessed through the Fisher’s Exact Test. Significance of semi-quantitative histological scores was determined with the Kruskal–Wallis Test with post-hoc analysis identifying differences between groups using the Conover method with the *p*-value adjusted according to the family-wide error rate procedure of Holm followed by the false-discovery rate procedure of Benjamini-Hochberg.

### Ethics approval and consent to participate

The husbandry and experimental protocols were approved by our Toronto General Hospital institutional research ethics board. The care of these animals followed recommendations from the *Principles of Laboratory Animal Care* by the National Society for Medical Research and the *Guide for the Care of Laboratory Animals* by the National Institutes of Health.

## Results

### Animal demographics and surgical procedure

Weight of pigs utilized in autotransplantation did not differ between groups (HBD: 33.3 ± 2.1 kg, DCD30min: 31.3 ± 1.2 kg, DCD60min: 29.5 ± 1.3 kg, DCD90min: 30.7 ± 0.7 kg and DCD120min: 31.0 ± 1.4 kg, *p* > 0.05) Between the HBD, DCD30min, DCD60min, DCD90min, and 120 min groups, there were no differences in total cold storage time (475.3 ± 9.1 min, 492.4 ± 15.0 min, 513.3 ± 37.8 min, 487.3 ± 18.6 min, and 517.5 ± 14.5 min, *p* = 0.08) and total vascular anastomosis time (36.7 ± 10.0 min, 33.8 ± 7.1 min, 47.7 ± 13.6 min, 44.0 ± 6.0 min, and 42.0 ± 10.1 min, *p* = 0.32).

### Recipient survival

**Figure 2:**
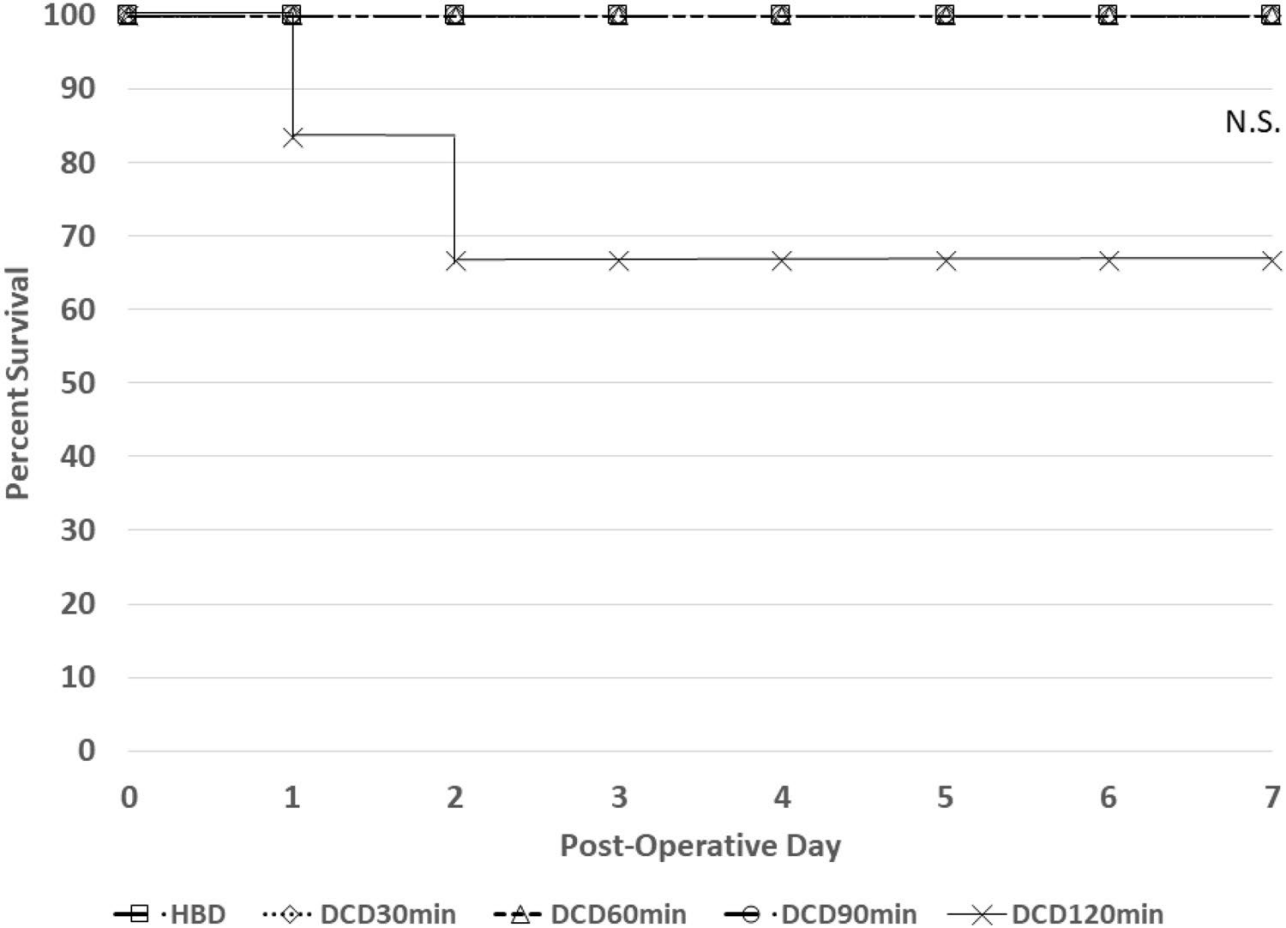
Recipient survival following transplantation of grafts subjected to no warm ischemia (HBD) or progressive warm ischemia of 30, 60, 90, or 120 min followed by 8 h of static cold storage. Survival is depicted in a Kaplan–Meier plot. *HBD* heart beating donor, *DCD* Donation after Cardiac Death. *HBD* heart beating donor. *N.S.* Not significant.

All recipients of kidneys in the HBD, DCD30min, DCD60min, and DCD90min group survived the 7-day observation period. Conversely, 2/6 animals that received kidneys in the DCD120min group required early euthanasia (Fig. 2). One was sacrificed on POD1 due to significant lactic acidosis. The second experienced respiratory distress and was sacrificed on POD2 with necropsy findings of bilateral hydrothorax (*p* = 0.28).

### Incremental renal function impairment with increasing WI

**Figure 3:**
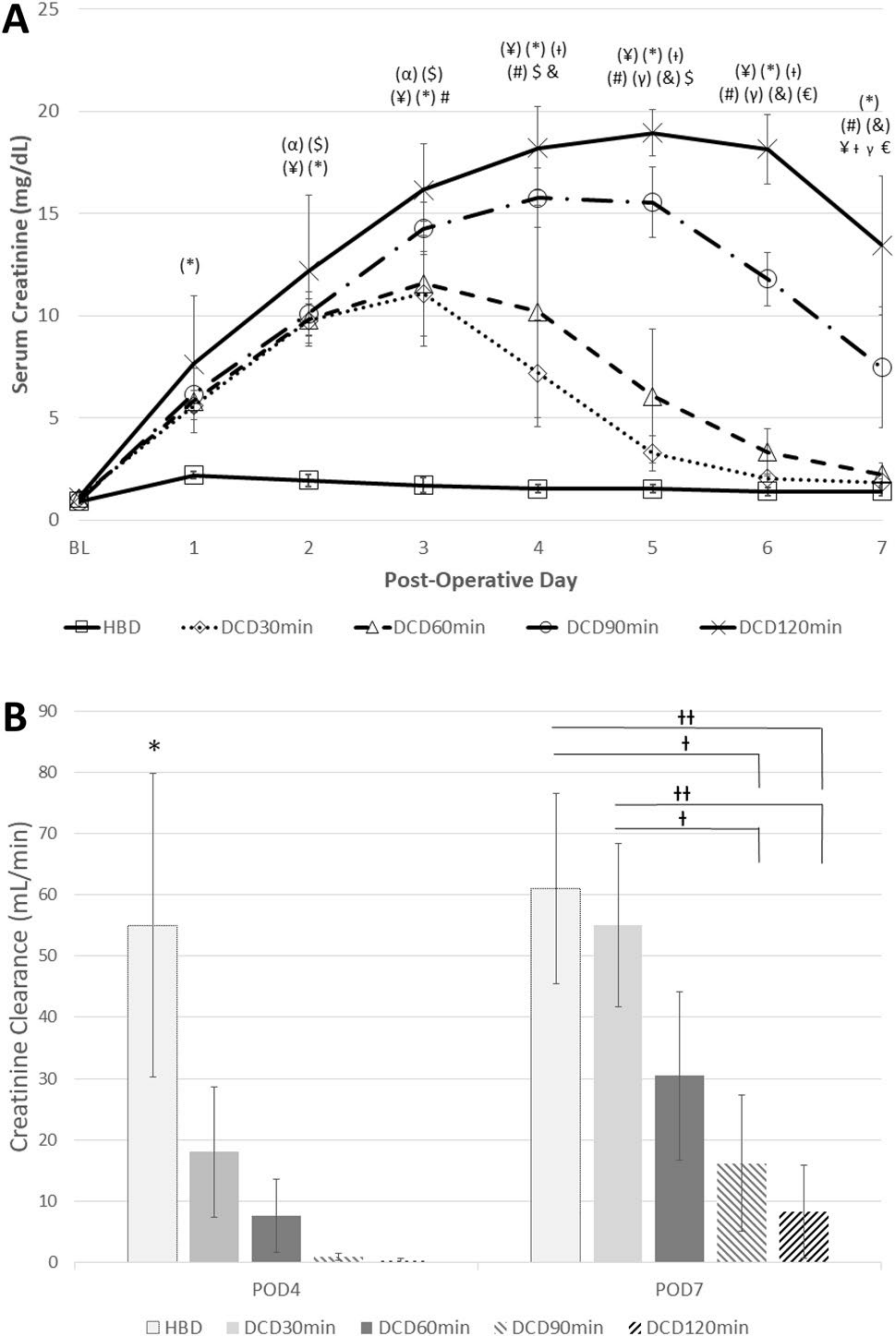
Post-operative renal graft function measured through (A) serum creatinine expressed as mean  +/− standard deviation [Statistical difference *p* < 0.05 with α: HBD vs DCD30min, $: HBD vs DCD60min, ¥: HBD vs DCD90min, *: HBD vs DCD120min, †: DCD30min vs DCD90min, #: DCD30min vs DCD120min, γ: DCD60min vs DCD90min, &:DCD60min vs DCD120min, €: DCD90min vs DCD120min. In brackets: *p* < 0.01], and (**B**) creatinine clearance obtained following 24 h urine collection expressed as mean  +/− standard deviation. *p < 0.05 between HBD and all groups. †*p* < 0.05 and ††*p* < 0.01 between defined groups. *HBD* Heart Beating Donor, *DCD* Donation-after-Cardiac Death, *POD*: Post-Operative Day.

Renal function, as measured by SCr, demonstrated an incremental worsening with increasing WI injury prior to procurement. The SCr peaked earlier and was lower in the HBD group (POD1: 2.2 ± 0.2 mg/dL) compared to the DCD30min (POD3: 11.1 ± 2.1 mg/dL), DCD60min (POD3: 11.6 ± 3.1 mg/dL), DCD90min (POD4: 15.8 ± 1.4 mg/dL) and DCD120min (POD5: 19.0 ± 1.1 mg/dL) groups (*p* < 0.01) (Fig. 3a). The SCr peaks were significantly different between the DCD90min and DCD120min groups (*p* = 0.01). By the end of the observation period, the SCr remained most elevated in the DCD120min group (13.5 ± 3.4 mg/dL) compared to the HBD (1.4 ± 0.2 mg/dL, *p* = 0.002), DCD30min (1.8 ± 0.2 mg/dL, *p* < 0.001), and DCD60min (2.2 ± 0.6 mg/dL, *p* = 0.003). POD7 SCr also trended higher in the DCD120min group compared to the DCD90min (7.5 ± 3.0 mg/dL, *p* = 0.06). However, the decrease in SCr on POD7 from its peak in the DCD120min group was significant (*p* = 0.03) (Fig. 3a).

CrCl also demonstrated worsening renal function with WI prior to kidney retrieval (Fig. 3b). CrCl on POD4 was the highest in the HBD group (55.0 ± 24.7 ml/min, *p* < 0.01). Although significance was not reached between groups with increasing WI, a trend of decreasing CrCl with increasing WI was observed at this timepoint (DCD30min: 18.0 ± 10.6 ml/min, DCD60min: 7.6 ± 5.9 ml/min, DCD90min: 1.0 ± 0.6 ml/min, DCD120min: 0.3 ± 0.3 ml/min, *p* = 0.23–0.90). Similar findings were observed on POD7 CrCl with the HBD group and DCD30min group (61.0 ± 15.6 ml/min and 55.0 ± 3.4 ml/min, respectively) significantly higher than the DCD90min group (16.2 ± 11.1 ml/min, *p* < 0.05) and DCD120min group (8.2 ± 7.6 ml/min, *p* < 0.01). The trend of decreasing CrCl with increasing WI corresponds to the finding of increasing SCr.

### SRD occurs in DCD grafts exposed to 120 min WI

**Figure 4:**
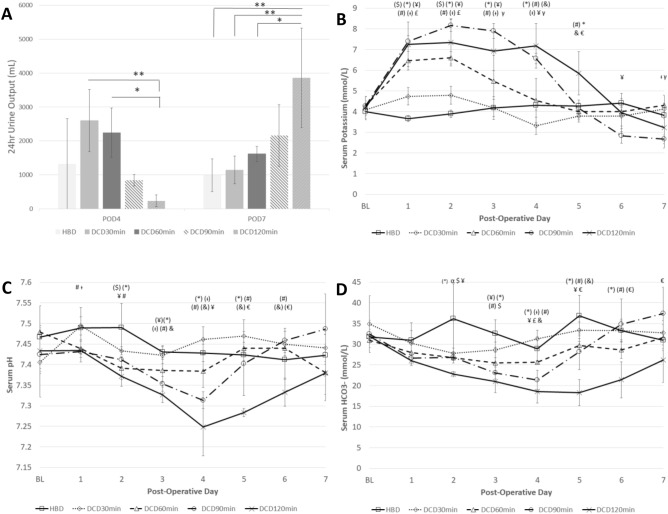
Biochemical characteristics of HBD and DCD kidneys (**A**) POD4 and 7–24 h urine output (mean  +/− standard deviation, *p < 0.05 **p < 0.01). (**B**–**D**) Post-operative serum electrolyte and acid–base regulation including (**B**) Serum Potassium. (**C**) Serum pH and (**D**) Serum bicarbonate levels. Values are expressed as mean +/− standard deviation. Statistical difference *p* < 0.05 with α: HBD vs DCD30min, $: HBD vs DCD60min, ¥: HBD vs DCD90min, *: HBD vs DCD120min, £: DCD30min vs DCD60min, †: DCD30min vs DCD90min, #: DCD30min vs DCD120min, γ:DCD60min vs DCD90min, &:DCD60min vs DCD120min, €:DCD90min vs DCD120min. In brackets: *p* < 0.01. *POD* Post-Operative Day, *pH* power of Hydrogen, *HCO3* Bicarbonate, *HBD* Heart Beating Donor, *DCD* Donation after Cardiac Death.

Signs of SRD were only present in the DCD120min group. This included oliguria with < 500 mL of urine in the 24 h urine collection completed from POD3-4. All animals in the DCD120min group had a 24 h UO < 500 mL (235 ± 172 mL). This was significantly different than the volume collected in the DCD30min group and the DCD60min group (2597 ± 916 mL *p* < 0.01 and 2245 ± 731 mL *p* < 0.05, respectively). Although significance was not reached between the other groups, no other group apart from the DCD120min group had an animal with < 500 mL of urine in the 24 h collection (Fig. 4a).

In the context of oliguria, dialysis would be indicated with a concurrent potassium abnormality. Potassium was measured in serum and POD4 values were considered as corresponding 24 h UO was available. Elevated serum potassium above 6.0 mmol/L was present in all 4 surviving animals of the DCD120min group (7.2 ± 1.1 mmol/L). This was significantly elevated compared to HBD (4.3 ± 0.2 mmol/L, *p* < 0.01), DCD30min (3.3 ± 0.4 mmol/L, *p* < 0.01), and DCD60min (4.5 ± 1.1 mmol/L, *p* < 0.01). There was no statistical difference between the DCD120min and DCD90min group (6.6 ± 0.3 mmol/L, *p* > 0.05) at this timepoint (Fig. 4b).

Refractory acidosis in the context of low UO could be considered as an indication for dialysis. The serum pH was assessed on POD4 and found to be significantly acidotic in the DCD120min group (7.25 ± 0.07) compared to all but the DCD90min group (HBD: 7.43 ± 0.02, *p* < 0.01, DCD30min: 7.46 ± 0.03, *p* < 0.01, DCD60min: 7.39 ± 0.04, *p* < 0.01, and DCD90min: 7.31 ± 0.02 *p* > 0.05, respectively). This trend continued on POD5 and was also observed compared to all groups that received WI on POD6 (Fig. 4c). This acidosis has a metabolic component with lower serum bicarbonate in the DCD120min group at POD4 (DCD120min 18.6 ± 2.7 mmol/L) compared to all groups except the DCD90min (HBD: 28.9 ± 2.9 mmol/L, *p* < 0.01, DCD30min: 31.3 ± 2.1 mmol/L, *p* < 0.01, and DCD60min: 25.6 ± 0.4 mmol/L, *p* < 0.05) (Fig. 4d).

All 4 surviving animals in the DCD120min group fulfilled our definition of SRD with a 24 h UO < 500 mL from POD3-4 and a concurrent serum potassium > 6.0 mmol/L. No other animal had SRD by this definition (*p* = 0.003). Although all animals in the DCD90min group had serum potassium above 6.0 mmol/L on POD4, they demonstrated acceptable UO above 500 mL for 24 h from POD3-4. No other animals in any other group had a serum potassium above 6.0 mmol/L or a POD3-4 UO less than 500 mL.

By the end of the observation period on POD7, UO improved in the DCD120min grafts. Potassium also decreased and was not statistically different from other groups (3.2 ± 1.0 mmol/L *p* = 0.16–0.72). No kidneys in the DCD120min group or any other group would fulfill the criteria for DGF at POD7.

### Increased tubular injury is evident in grafts that experience DGF

**Figure 5:**
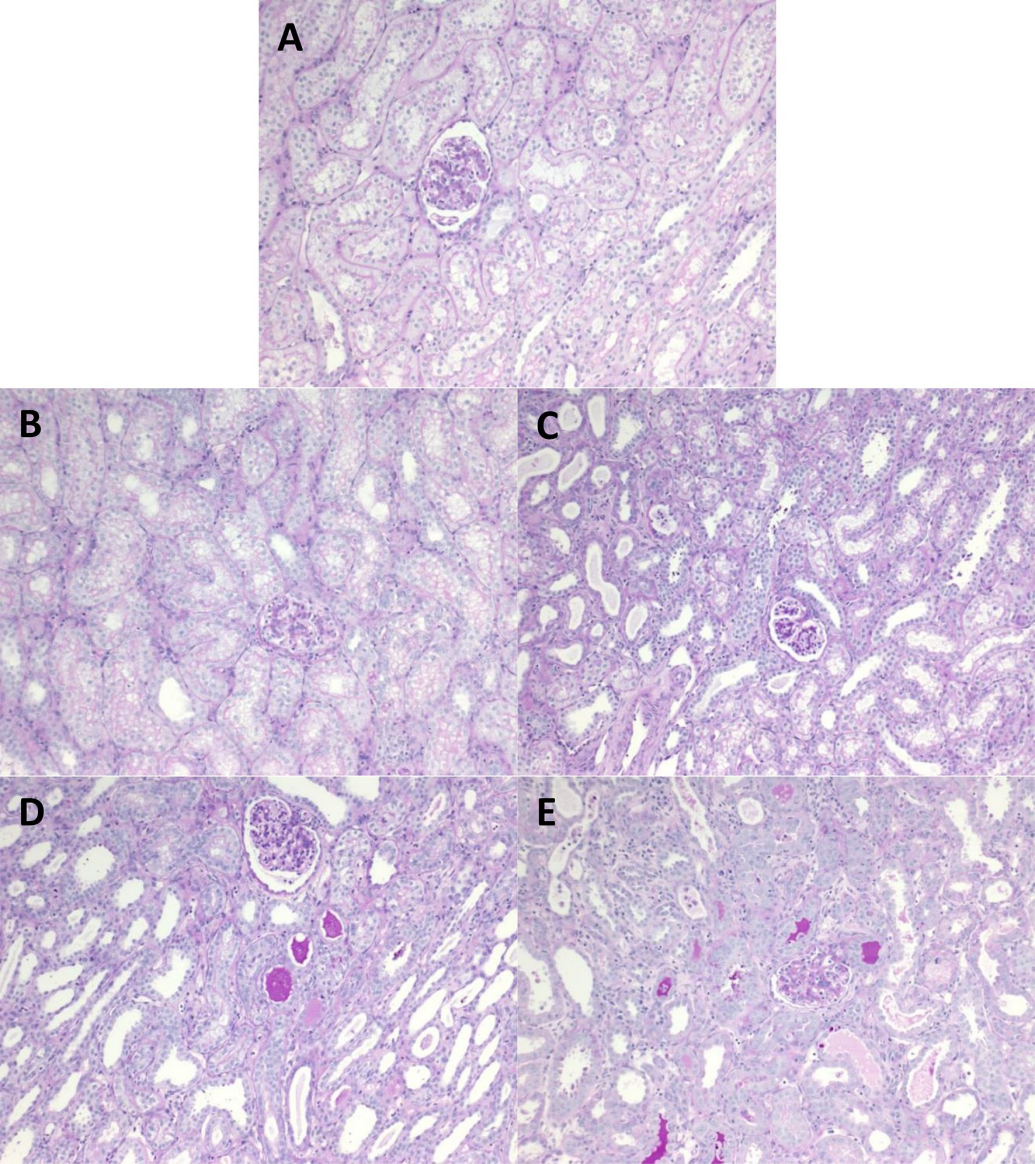
Representative periodic-acid-Schiff (PAS) staining. Post-operative day 7 specimens were collected of kidneys subjected to (**A**) no warm ischemia (heart-beating donor [HBD] group), or (**B**) 30 min (DCD30min), (**C**) 60 min (DCD60min), (**D**) 90 min (DCD90min), or (**E**) 120 min (DCD120min) of warm ischemia prior to transplantation. 10 × magnification.

Renal biopsies taken at the time of sacrifice on POD7 showed that increased exposure to WI prior to procurement correlated with increased histopathologic injury with more brush border loss, tubular dilation, epithelial vacuolation, and luminal debris. There was significantly more injury in the DCD120min group, which also demonstrated DGF, with a median tubular injury score of 3.0 (range 3.0–3.0) compared to all other groups except the DCD90min group (HBD: median score 0.5 [range 0.0–0.5] *p* < 0.01, DCD30min: median score 0.5 [range 0.5–1.0] *p* < 0.01, and DCD60min: median score 1.0 [range 1.0–2.0] *p* < 0.01). Although significance was not reached, the trend was towards worse injury in the DCD120min group compared to the DCD90min group (median score 2.0 [range 1.5–3.0] *p* = 0.08). There were also significantly more inflammatory infiltrates in biopsies from the DCD120min group (median score 2.0 [range 1.0–2.0]) compared to the HBD (median score 0.5 [range 0.5–1.0] *p* < 0.05), DCD30min (median score 0.5 [range 0.0–1.5] *p* < 0.05), and DCD60min (median score 0.5 [range 0.5–1.0] *p* < 0.05) groups (Table 1, Fig. 5).

**Table 1:** Worsening histological tubular injury and inflammation with increasing warm ischemia. **A** Histological scores of tubular injury (0–3) and inflammation (0–3) assessed by a renal pathologist blinded to the groups. Scores taken at time of sacrifice on post-operative day 7 between all groups. Statistical difference *p* < 0.05 with $: HBD vs DCD60min, *:HBD vs DCD120min, #:DCD30min vs DCD120min, In brackets: *p* < 0.01. *HBD* Heart beating donor. *DCD* Donation after cardiac death. **B** Tubular injury and **C** inflammatory semiquantitative scores were determined using these parameters.

A	POD7	
	Median Tubular Injury Score (Range)	Median Inflammation Score (Range)
HBD	0.5 (0.0–0.5)	0.5 (0.5–1.0)
DCD30MIN	0.5 (0.5–1.0)	0.5 (0.0–1.5)
DCD60MIN	1.0 (1.0–2.0)(*)(#)	0.5 (0.5–1.0)
DCD90MIN	2.0 (1.5–3.0)(*)(#)	1.0 (1.0–1.5)
DCD120MIN	3.0 (3.0–3.0)(*)(#)($)	2.0 (1.0–2.0)*#$

	B. Tubular Injury Score	C. Inflammation Score
Criteria	Brush border loss	Number of infiltrating interstitial mononuclear cells averaged over 30 high powered fields
	Tubular dilatation	
	Epithelial vacuolation	
	Thinning and sloughing	
	Luminal debris

## Discussion

DGF is an important clinical entity following DCD kidney transplantation. Its etiology and the pathophysiological mechanisms that lead to its long-term consequences are not well delineated. This is due in part to the lack of mechanistic studies utilizing animal transplantation survival models.

In this report, we addressed this shortcoming using a porcine DCD autotransplantation model with kidney grafts procured following incremental increases in WI times. We demonstrated progressive deterioration of renal function with increase in WI times, however only animals exposed to 120 min of WI demonstrated SRD that resembled DGF clinically. We did not utilize definitions of SRD that were based on SCr rise since all otherwise healthy porcine recipients would be expected to have optimal renal function and low baseline creatinine prior to nephrectomy and autotransplantation of DCD grafts. A rise in SCr would be expected in this setting. Instead, we utilize a commonly accepted definition of DGF which is the indication for dialysis in the first week following transplantation. Moreover, our findings were inconsistent with PNF as improving renal function was observed by POD7. As no other animals apart from the surviving pigs in the DCD120min group demonstrated these characteristics, the DCD120min group thus represents a robust and clinically relevant model of SRD that resembled clinical DGF.

Rodent models have previously been used to study renal function following induction of WI injury. These models of acute kidney injury have been primarily performed through clamping of the renal vascular pedicle and subsequent unclamping after a time interval^17–20^. However, graft injury that occurs through procurement, storage, and transplantation are not assessed in these models. Fewer studies have utilized rodent kidney transplantation models to mimic human transplantation^21,22^. Nevertheless, important limitations in these rodent transplantation models exist including no established model of DGF and the homogenous genetic background that may preclude the generalizability of findings. Furthermore, technical differences exist due to the size of the organisms that requires microsurgical expertise that are not widely available. The size may also limit the assessment of some treatment strategies aimed to reduce preservation injury such as perfusion technologies that are not easily scalable with fidelity to the clinical setting^23,24^.

Porcine kidney DCD autotransplantation models have generated considerable interest to overcome many of these limitations. The effects of increasing WI injury in a DCD model in this setting has been previously reported. Hosgood et al*.* induced WI for 7–120 min through renal vessel clamping prior to retrieval and subsequently stored in static cold conditions for 2 hrs^25,26^. In these studies, renal function was then assessed through an *ex-vivo* warm perfusion system with an erythrocyte-based perfusate to simulate transplantation. An incremental decrease in renal hemodynamics, CrCl, UO, and fractional excretion of sodium were observed with prolonging WI times. However, extrapolating these findings from *ex-vivo* reperfusion are difficult as important mediators of post-transplantation injury are not present. Moreover, longer-term graft function indicators are not assessed. As such, survival models are required to determine the presence, mechanism, and treatment of DGF.

Current porcine DCD autotransplantation model protocols have resulted in acceptable post-operative renal graft function. Our group has previously utilized 30 min WI in these DCD models with 8–16 h of SCS with follow-up of 7–10 days^11,27–29^. While SCr peaks remained lower than those observed in the DCD120min model, importantly the early indications for dialysis were not observed. Other groups have utilized 30 min DCD models with varying cold storage times with similar results even in allogenic settings^30,31^.

Some groups have extended the WI time. Seventy-five min of WI with 16 h of cold storage was utilized by Lohmann et al*.* in a study to assess animal welfare following prolonged WI. Although peak serum potassium of 6.8 mmol/L was observed in 1/2 of these pigs, a lack of 24 h urine collection precludes the determination of DGF in this setting^32^. In a study examining the effects of argon and xenon inhalation after transplantation, De Deken, et al*.* utilized 60 min of WI and 18 h of cold storage. Control animals without treatment demonstrated POD3 UO with a large interquartile range up to 90 mL/h. Without serum electrolytes reported, this UO suggests most animals at this timepoint were not experiencing DGF by our definition^33^. Similarly, 60 min of WI and 24 h of preservation were reported by Thuillier et al*.* and although UO was less than 300 mL in 4/12 pigs on POD4 no electrolyte abnormality to indicate a need for dialysis were reported^34^. Similarly, pathophysiological mechanisms of IRI and different treatment modalities for IRI were studied utilizing extended warm and cold ischemic times in porcine autotransplantation models, although clinical parameters that would resemble DGF were likewise not explicitly defined^24,35–38^. Finally, a study reported a porcine transplantation into allogenic recipients with minimal immunosuppressants after 60 min of WI and extracorporeal perfusion. A definition of DGF was given a SCr > 5 mg/dL on any post-operative day^39^. This model would be difficult to replicate widely, and the definition would be insufficient to indicate dialysis in our experience. To our knowledge, DGF in a porcine DCD survival model that is reproducible has not been otherwise described previously.

An induced model of DGF was described in the porcine setting by Keller et al*.* where porcine kidney vessels were clamped for 2 h after transplantation. Although this set up may be useful to develop technologies that can diagnosis DGF development, this artificial protocol is less applicable to the study of mechanisms and treatment of DGF^40^. A transplantation survival model demonstrating DGF is essential for this purpose.

Finally, Brasile et al*.* described a model of PNF following 120 min of WI in experiments using canine kidney autotransplanation. Kidneys were stored for either 18 h or 24 h in cold stored groups (n = 2 per control group) and all these control animals required earlier euthanasia due to poor overall health and rising SCr^41,42^. These kidneys were considered to represent PNF although it is unclear if renal function would improve if animals could be observed for longer time periods. It is also possible that canine kidneys are more susceptible to WI damage.

Mimicking DCD conditions with 120 min of WI prior to procurement was essential for the SRD demonstrated in this model that resembled DGF. In these ischemic environments, the switch to anerobic metabolism to produce energy is thought to initiate the events that result in DGF. Lactate accumulates promoting an acidotic environment. This then cause enzymatic dysfunction leading to altered intra- and extra-cellular electrolyte concentrations promoting necrosis and apoptosis^43^. Reperfusion returns aerobic metabolism and increases the levels of reactive oxygen species that further cause cellular damage. These products of cell damage cause endothelial activation and initiation of innate and adaptive immune responses. The activation of pathways leading to fibrosis are thought to contribute to long-term graft dysfunction^43^. These responses would likely be more potent following prolonged WI injuries, such as in the DCD120min model thus producing SRD only in this group. Although the mechanisms that led to the need for euthanasia in 2 of the DCD120 min model remain speculative, it is conceivable that the products of cellular damage were sufficiently present that upon reperfusion a systemic inflammatory response syndrome occurred leading to further metabolic derangement and multi-organ system failure^44,45^. Importantly, the difference in survival did not reach statistical significance although this study was not designed to be sufficiently powered to identify such end-point. This model that we describe provides a useful platform to study the effects of DCD conditions, IRI, and the downstream sequelae that leads to SRD that is similar to clinical DGF.

Important limitations of this model must be acknowledged. First, these WI conditions are not entirely reminiscent of human DCD donors, as human donors typically experience significant comorbidities and heterogeneity in the agonal phase that cannot be replicated in otherwise healthy swine. The impact of alloimmunity potentiating any effects of IRI is also not assessed in this model. The absence of these conditions may underestimate the incidence and effects of DGF that would occur with similar WI insults. Moreover, our results may underestimate the true extent of injury due to the selection bias that occurred by the exclusion of animals that were euthanized. However, this model likely leads to more damage than would occur over a similar agonal phase time in clinical settings as vascular clamping completely prevents perfusion of the graft. Long-term complications of DGF were also not assessed.

To our knowledge, this work is the first to describe the effects of prolonged WI time in a clinically relevant survival model of renal transplantation. It is also the first to describe a robust model of SRD that resembles the clinical development of DGF. This provides a useful platform to better understand the pathophysiological consequences of WI present in DCD conditions, IRI, and how these lead to the development of SRD/DGF. The longer-term complications can also be assessed in the future. Finally, treatments aimed at ameliorating or preventing the development of DGF, including the assessment of different perfusion storage technology, can be tested in this model that more closely mimics clinical conditions.

## Data Availability

The datasets during and/or analysed during the current study available from the corresponding author on reasonable request.

## Abbreviations

CrCl: Creatinine clearance
DCD: Donation after cardiac death
DGF: Delayed graft function
ESKD: End-stage kidney disease
HBD: Heart-beating donor
HTK: Histidine-tryptophan-ketoglutarate
IV: Intravenously
PAS: Periodic acid-Schiff
PNF: Primary non-function
POD: Post-operative day
SCr: Serum creatinine
SCS: Static cold storage
UO: Urine output
WI: Warm ischemia
WMS: Withdrawal of mechanical supports
